# Simpatico: accurate and ultra-fast virtual drug screening with atomic embeddings

**DOI:** 10.1101/2025.06.08.658499

**Published:** 2025-06-08

**Authors:** Jeremiah Gaiser, Travis J. Wheeler

**Affiliations:** School of Information, University of Arizona, Tucson, AZ 85721; College of Pharmacy, University of Arizona, Tucson, AZ 85721

## Abstract

Building on established methods for molecular docking, structure-based deep learning has recently yielded important advances in virtual drug screening. We present simpatico, a method that follows an alternate approach, based on the field of Representation Learning, to dramatically speed the process of accurate drug screening. Simpatico employs graph neural networks to produce high-dimensional embeddings for the atoms of proteins and small molecules, and uses these embeddings to rapidly produce accurate predictions of the interaction potential for drug candidates with target protein pockets. Simpatico can search a database containing 600 million drugs for good binding candidates to a single protein pocket in 2.5 hours on a single GPU. Despite being >1000x faster than state of the art docking and diffusion-based methods, simpatico is competitive with the most accurate of those methods. We also observe that simpatico embeddings can be used to explore toxicity risk and to identify proteins with similar binding potential. Simpatico is open source software; all code, weights, and data may be accessed at https://github.com/TravisWheelerLab/Simpatico.

## 1 INTRODUCTION

The challenge of predicting whether a small molecule drug will bind to a target protein pocket is a cornerstone of virtual drug screening [Sadybekov and Katritch, 2023], a critical problem in drug discovery. Traditionally, the field has been dominated by “docking” methodologies [Jones et al., 1997, Friesner et al., 2004, Eberhardt et al., 2021], which explore the vast space of potential binding configurations in search of a pose with favorable energetics, supporting likely binding interaction. Most recent improvements to docking accuracy have resulted from alternative methods for assigning score to the myriad poses explored during docking, shifting away from physics-based energetic equations and towards learned interaction fingerprints [Marcou and Rognan, 2007, Desaphy et al., 2013] or sophisticated deep learning approaches [Shen et al., 2022, 2023, Zhang et al., 2023, Moon et al., 2024]. Recent advances in diffusion-based approaches [Corso et al., 2022, Abramson et al., 2024, Cao et al., 2025] offer an alternative to classical pose enumeration approaches, with promising potential.

The recent appearance of massive libraries of synthesizable compounds [Enamine, 2022, Tingle et al., 2023] has motivated the development of computationally efficient methods to sift through millions or even billions of molecules to identify promising drug candidates for a protein target. Generally, docking- and diffusion-based prediction of the potential for a small molecule to bind to a target pocket requires time measured in the order of (at least) seconds, too slow for billion-scale compound search. Here we describe a simple graph neural network model that is designed to very rapidly compute a measure of compatibility between a small molecule and a target protein pocket. The tool implementing this model, called simpatico (simple atomic interaction-prediction with contrastive learning), produces high-dimensional embeddings (representations) of protein pockets and candidate drugs, then rapidly identifies good binding candidates by searching for near neighbors using an efficient vector database. Specifically: simpatico accepts protein structures (as from PDB [Burley et al., 2023]) and small-molecule descriptions (SMILES [Weininger, 1988]) as input, produces unique embeddings for each individual atom in protein pockets and small molecule compounds, then uses these embeddings as the means of computing an overall measure of the binding potential between a pocket-compound pair.

To learn a useful embedding scheme, Simpatico uses a training scheme based on contrastive-learning to parameterize two graph neural network (GNN) models that separately represent proteins and ligands. Simpatico was trained with protein-ligand interaction data from PDBBind [Liu et al., 2015], and efficiently maps protein and ligand atoms into a rich semantic embedding space that is characterized by a useful property: the high-dimensional embedding of a protein atom is expected to be close to the embedding of a small molecule atom if those atoms are observed interacting in real-world bound structures.

In the context of binding, we show that simpatico’s distance property, combined with a simple method for accumulating binding support across multiple atomic relationships, enables rapid and accurate estimation of the potential for a ligand to bind to a given protein pocket. In a mock screen for actives in a large database of random molecules (~600M), simpatico demonstrates high enrichment at very small sampling ratios, with some targets achieving enrichment values of several thousand fold, while requiring only ~14 seconds per million small molecules for a typical protein pocket target. When applied to challenging decoy screening tasks, simpatico outperforms energy based methods such as Autodock Vina, is comparable in accuracy to newer Deep Learning approaches, and is faster than both by many orders of magnitude. Beyond binding prediction, we show that simpatico embeddings may be useful in the identification of attractive protein receptors for a specific ligand target, a task with direct application in the field of toxicology and study of off-target effects. Simpatico is open source software; all code, weights, and data are available at https://github.com/TravisWheelerLab/Simpatico.

### 1.1 Graph Neural Networks for molecules

Graph neural networks (GNNs) are deep learning architectures designed to operate directly on graph-structured data, learning to update node features iteratively through multiple message-passing layers. This design allows GNNs to model data with non-Euclidean relationships, making them ideally suited for molecular structures. They naturally represent chemical structures through a node-edge framework, wherein atoms correspond to nodes and chemical bonds correspond to edges. See [Sanchez-Lengeling et al., 2021] for a practical introduction to GNNs.

### 1.2 Contrastive Learning

Contrastive learning is a self-supervised learning technique widely used in representation learning. The basic strategy of contrastive learning is to update network parameters in a way that pulls representations closer together in embedding space if their original inputs belong to the same underlying class and pushes them apart if their original classes differ. Over many training batches, the network learns a structured embedding space in which underlying classes can be distinguished. To achieve this, the network must learn a rich set of features corresponding to specific classes.

### 1.3 Notation

For clarity, we introduce the following notation. A protein-ligand complex comprises a protein pocket and a small molecular compound (ligand). When it is necessary to distinguish unique pockets or compounds, we employ subscripts explicitly denoting their distinct identities. When discussing a collection of pockets P, we may refer to an individual pocket as $P_{m}$. Similarly, each $C_{n}$ belongs to a collection of ligands C. Proteins and ligands themselves consist of atoms, with $p_{m,i}$ representing the i-th atom in the m-th pocket from the set P, and $c_{n,j}$ representing the j-th atom in the n-th ligand from the set C. For notational convenience, when pocket/compound identity is clear, we remove the pocket/compound identifier, leaving P, C, $p_{i}$, and $c_{j}$.

A compound C might be known to associate with a specific pocket P. In other words, C is a binding partner or active compound of P. This relationship is denoted as C∼P, or equivalently, P∼C. If we know that compound C explicitly does *not* associate with protein pocket P, we write that C≁P.

For any pocket P and some known active C such that P∼C, the binding affinity between the two chemical structures will depend on the interactions between their atoms. We consider any two atoms $p_{i}$ and $c_{j}$ belonging to a protein P and compound C to be interacting if they are observed within 4 angstroms (Å) of one another in a resolved crystal structure of the complex PC. In shorthand, we express this interaction as $p_{i}∼c_{j}$ (or equivalently $c_{j}∼p_{i}$).

The above notation is useful for describing physical relationships among protein pockets, small molecular compounds, and their constituent atoms. The aim of simpatico is to train an encoding function f(⋅∣θ) to project these atoms into a high-dimensional embedding space $R^{d}$. To differentiate an atom $p_{i}$ or $c_{j}$ from its embedding representation, we denote atom embeddings as $\vec{p_{i}}\in R^{d}$, such that $f(p_{i}|\theta )=\vec{p_{i}}$ (and similarly, $f(c_{i}|\theta )=\vec{c_{i}}$). The standard notation for describing the set of k nearest neighbors for some vector $\vec{p_{i}}$ in high dimensional space is $N_{k}(\vec{p_{i}})=\{x_{1},x_{2},\ldots ,x_{k}\}$, where each $x_{l}$ is one of the k closest points to $\vec{p_{i}}$ according to a specified metric (in this case, the Euclidean ($L_{2}$) distance). If an embedding $\vec{c_{j}}$ belongs to the set of k nearest neighbors $N_{k}(\vec{p_{i}})$, we write $\vec{c_{j}}∼\vec{p_{i}}$.

When we discuss the collection of atomic embeddings of all the atoms in some pocket P or compound C, we will refer to $\vec{P}$ or $\vec{C}$. To round out our notation, we will refer to a collection of $\vec{P}$ or $\vec{C}$ with $\vec{P}$ or $\vec{C}$, respectively.

## 2 METHODS

### 2.1 Motivation

The simpatico approach is based on the concept of a desirable semantic space for atomic embeddings of proteins and small molecules, where the space is defined by the characteristic that if a small molecular compound atom $c_{j}$ and a protein atom $p_{i}$ from a resolved protein-ligand complex are neighbors in 3D space ($c_{j}∼p_{i}$), then they should also be neighbors in the learned embedding space ($\vec{c_{j}}∼\vec{p_{i}}$). With a trained model, atomic representations in this space are implicitly organized according to some notion of physicochemical complementarity, or the likelihood that they interact with one another.

### 2.2 Data

Evaluation was performed using standard benchmarks: DEKOIS [Bauer et al., 2013], DUD-E [Mysinger et al., 2012], and LIT-PCBA [Tran-Nguyen et al., 2020] (see Supplementary Materials for descriptions of these data sets). To evaluate high-throughput speed and enrichment, these were supplemented by compounds from the Enamine REAL database [Enamine, 2022].

Training data were taken from PDBBind [Liu et al., 2015], a curated dataset of 19,443 high quality protein-ligand complexes spanning 3,876 distinct proteins, sourced from the Protein Data Bank [Burley et al., 2023]. PDBBind entries provide simplified (no ions, water or extraneous heteroatoms) structures of ligands in complex with their protein target.

Separate models were trained on two distinct train-validation splits. In the first, we removed from the PDBBind training data any protein-ligand *complexes* found in DUD-E and LIT-PCBA. This training set retained the vast majority of the complexes present in the original data, with just 113 holdouts. Though this ensures that the model is not trained on complexes that are part of the evaluation, some of the training data involves *proteins* represented in the test data (paired with different ligands). To assess Simpatico’s power to generalize to completely novel protein structures, we trained a separate model on a subset of PDBBind that did not include *any proteins* present in the DEKOIS dataset. This resulted in a significant reduction of training data: in total, 3,885 complexes (~20 % of the data in PDBBind) were withheld from the training set. To our knowledge other deep learning approaches were trained on *all* complexes in PDBBind, and are thus subject to data memorization.

### 2.3 Compound Graph Construction

**Figure 1:**
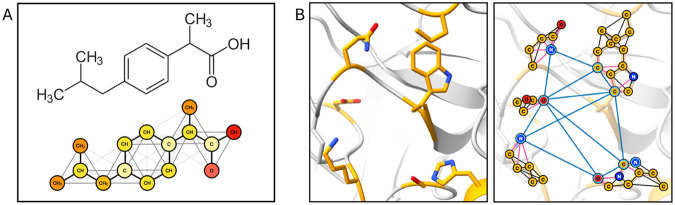
Graph Construction. In both compound graph and protein pocket graph, nodes represent heavy atoms. (A) In the compound graph, edges are created based on covalent bonds, representing one-hop, two-hop, and three-hop connections. (B) For a protein pocket graph: given a protein structure and a set of pocket surface atoms, edges are created in a neighborhood around predicted interacting surface atoms. See text for details.

Small molecule compounds (drugs) are converted to Pytorch Geometric (PyG)-formatted graphs [Fey and Lenssen, 2019]. Each heavy atom is represented by a node in the graph, with two features per node: (i) the atomic species and (ii) the number of attached hydrogen atoms (one-hot vector). Edges are established between any 2 nodes within 3 covalent bonds of one another. Edge features consist solely of a one-hot vector indicating whether an edge represents a 3-hop, 2-hop, or 1-hop edge, where a 1-hop edge is equivalent to a covalent bond (Figure 1A). For training data ligands, atomic positional data according to the protein-ligand complex is stored for convenience, but is never made available to the model.

### 2.4 Pocket Graph Construction

Simpatico’s algorithm requires specification of a target pocket. Given a full protein structure (PDB file), a 3-dimensional coordinate specifies the center of the pocket. This centroid can be used to determine the set of protein atoms that are considered to be part of the binding surface of the protein (‘pocket surface atoms’: $S=\{p_{i}\;|\;\forall \;i\;in\;pocket\;surface\;selection\}$, and S⊂P). This subset of the protein-atom point cloud is represented by the blue-outlined atoms in Fig. 1B. These are the atoms expected to possibly participate in binding interactions with a ligand. During training, we perform a randomized centering of a known pocket to avoid overdependence on precise knowledge of pocket center (see supplementary material). This process can be used during inference, but by default, our tests accept a known bound ligand structure (either from a solved complex or docking), designating any protein atom node within 4 angstroms of a ligand atom as a surface pocket atom (given some complex PC, $S=\{p_{i}\;|\;p_{i}∼c_{j}\;for\;some\;c_{j}\in C\}$).

With this set of surface atoms defined, a PyG-formatted graph is produced for the pocket, creating nodes for heavy atoms, and edges as follows:

For each pocket surface atom $s_{i}$, an edge is established between $s_{i}$ and the x surface atoms that are closest in 3-dimensional space ($E_{1}=\{s_{i}s_{j}\;|\;s_{j}\in N_{x}(s_{i})\}$; blue lines in Fig. 1B). Additionally, an edge is established between $s_{i}$ and the y nearest protein atoms, which do not necessarily belong to the set of surface atom nodes ($E_{2}=\{s_{i}p_{j}\;|\;p_{j}\in N_{y}(s_{i})\}$; pink lines in Fig. 1B). For each of these neighboring protein atoms $p_{j}$, an edge is established between $p_{j}$ and its closest z atom neighbors ($E_{3}=\{p_{j}p_{k}\;|\;p_{j}\in N_{y}(s_{i}),\;p_{k}\in N_{z}(p_{j})\}$; black lines in Fig. 1B). The final pocket graph is the union over these edges $E=E_{1}\cup E_{2}\cup E_{3}$, and N={p∣p∈E}.

This protein pocket graph design allows large-scale communication between likely interacting surface atoms, while sharing information locally among nearby atoms two levels deep.

### 2.5 GNN Architecture

Thus far, we have described the structure of the ligand and pocket atom graphs. These graphs are instantiated in two separate GNNs. All GNN model layers use Pytorch’s GATv2Conv, using multi-headed attention at each layer. The models are residually layered; each block consists of two GAT operations, the output of which is passed through a nonlinear SiLU layer and appended to the residual block input. This final value is passed through a small (p=0.1) dropout layer during training. Normalization layers are not used, as their inclusion was only observed to decrease performance.

For pocket graph model edge features, distances between adjacent atom nodes are passed through a single low-dimensional linear layer with ReLU activation. The output of this operation is used as the edge weight, which is the sole edge feature in the pocket GNN. Model details are provided in the supplementary materials.

### 2.6 Training

Throughout training, batches of b interacting protein-ligand pairs are sampled at random. For each sample in a training batch, the protein pocket associated with that pair is randomly sampled from the protein as sketched above and described in detail in S1, yielding the collection $PC=\{P_{q}C_{q}|1\leq q\leq b\}$ of protein pocket and compound graphs such that $P_{q}∼C_{q}$. Collections P and C are processed separately by their respective GNN encoders, producing pocket surface atom embeddings $\vec{P}$ and molecular compound atom embeddings $\vec{C}$. The resulting atom embeddings are then used as inputs in the following contrastive learning task.

Each $P_{q}C_{q}\in PC$ contains a collection of protein-ligand atom pairs such that $p_{q,i}∼c_{q,j}$. The embeddings from these pairs are used as anchor-positive pairs in the loss function described in the next section. For each positive pair ${\left[\vec{p_{q,i}},\vec{c_{q,j}}\right]}_{P}$, three negatives ${\left[\vec{p_{q,i}},\vec{c_{*,k}}\right]}_{N}$ are selected according to the following criteria, which are designed to prevent the model from learning spurious, trivial, or otherwise undesirable signals:

#### Random Negatives

For anchor-positive pair ${\left[\vec{p_{q,i}},\vec{c_{q,j}}\right]}_{P}$, some other pocket-compound pair $P_{r}∼C_{r}$ is selected at random from the same batch PC, and an interacting atom pair $p_{r,x}∼c_{r,y}$ is selected at random from that complex. The compound embedding from this positive pair is used as an anchor-negative pair ${\left[\vec{p_{q,i}},\vec{c_{r,y}}\right]}_{N}$. Interacting atoms are used as negatives to prevent the model from relying on signals that suggest non-activity, and instead require that the model distinguish interactions between active protein atoms and interactive but otherwise incompatible ligand atoms.

#### Self Negatives

Given the anchor-positive pair $[\vec{p_{q,i}}$, ${\vec{c_{q,j}}}_{P}$, a negative pair is produced by selecting a different atom embedding from compound $c_{q}$, yielding ${|\vec{p_{q,i}},\vec{c_{q,k}}|k\neq j]}_{N}$. $c_{q,k}$ is drawn from compound atoms that are among the 25% most distant ligand atoms from $p_{q,i}$. The use of self-negatives prevents the collapse of small compound node representations to the same value. Consequentially (and helpfully), this forces the models to truly distinguish the chemical environments between neighboring regions of pocket and small molecule alike, resulting in more finely grained representations of pockets and compounds.

#### Hard Negatives

For anchor-positive pair ${\left[\vec{p_{q,i}},\vec{c_{q,j}}\right]}_{P}$, a hard-negative compound $C_{h}$ is selected from separate small molecules in the batch PC such that $P_{q}≁C_{h}$, but $\vec{p_{q,i}}∼\vec{c_{h,k}}$, producing anchor-negative pair ${\left[\vec{p_{q,i}},\vec{c_{h,k}}\right]}_{N}$. In other words, a hard negative is an atomic embedding that does not belong to the positive ligand, but nevertheless is very close to the protein atom anchor in embedding space. Hard negatives are selected according to an increasingly ‘difficult’ schedule. Initially, a ‘hard’ negative is randomly selected from among all possible embeddings except the 1% most distant from $\vec{p_{q,i}}$; over time the radius of candidates for random sampling is reduced, so that eventually all hard negatives are sampled from only the 5% of embeddings closest to $\vec{p_{q,i}}$. This sampling radius diminishes after each epoch, decreasing linearly until locking in the final percentile at the 50th epoch.

Thus far, we have described a training scheme wherein the anchor embedding always corresponds to a pocket atom, and the positive/negative embeds to small compound atoms. These roles may be trivially reversed, and a small molecule atom may serve as an anchor to positive/negative embeds from a protein pocket. In simpatico’s training procedure, this role reversal is performed on alternating batches.

### 2.7 Loss Function

The simpatico loss function is a variation of the triplet margin loss function. Let a be the *batch anchor embedding* and let + be the *positive embedding*, where these are respectively the embedding of a protein atom and compound atom in an interacting pair $p_{i}c_{j}$. Then let – be a *negative embedding*, the embedding of some compound atom that does not interact with $p_{i}$. See the previous section for methods for selecting negatives.

**Figure 2:**
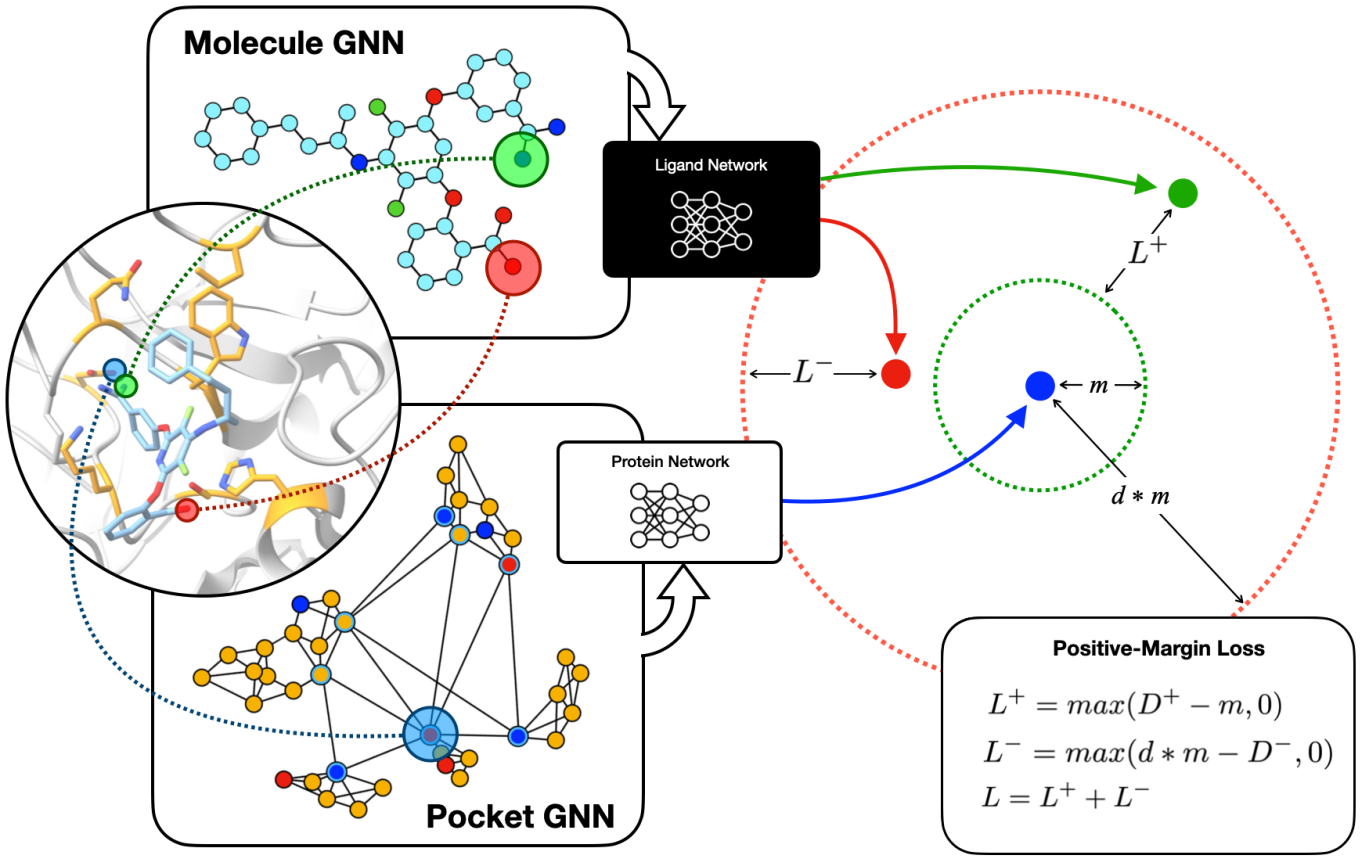
Loss Function. Following a modified triplet loss function framework, one pocket atom (‘anchor’) is embedded with the protein GNN, its interacting compound atom (‘positive’) is embedded through the molecule GNN, and one atom that does not interact with the anchor (‘negative’) is also embedded through the molecule GNN. The *positive margin loss* function encourages the positive embedding to achieve distance at most m from the anchor, while encouraging the negative embedding to achieve distance at least n. See text for details.

In our initial efforts with contrastive learning, we used the standard triplet margin loss function:

$$L(a,+,-)=max\{D^{+}-D^{-}+m,0\}$$

where $D^{+}$ is the anchor-positive embedding distance, $D^{-}$ is the anchor-negative embedding distance, and m is some margin value, usually 1. While triplet margin loss tends to succeed at minimizing the loss function by projecting anchor embeddings closer to positives than to negatives, this difference is only relative; we found that generally, while $(D^{+}-D^{-})>m$, $D^{+}>>m$ and $D^{-}>>m$, meaning that the loss function was minimized, but the goal of moving anchor embeddings close to positive embeddings was not reached. In order to enforce an embedding space such that complementary atomic embeddings are proximal in absolute distance, we devised the loss function that we refer to as *positive margin loss*, which is the sum of two loss values for positive and negative anchor-embed pairs ($L^{+}$ and $L^{-}$ respectively). See Figure 2.

The positive margin loss is minimized by projecting positives within some *absolute* distance of the anchor, and negatives at least some absolute distance away from the anchor.

One important benefit of this approach can be seen in the following example. Consider a single protein that is known to bind with multiple compounds. From this protein, a single pocket atom $p_{i}$ might interact with multiple compound atoms $c_{j}$ in these many binding events. With the positive margin loss, it is possible for each $c_{j}$ to be embedded such that the positive training loss $L^{+}$ for that pair is zero, without requiring that all $\vec{c_{j}}$ are identical.

### 2.8 Computing a score for a candidate pocket-ligand pair

In order to compute a scoring function $(P,C)\to R^{+}$ for the binding potential between a protein pocket P and drug candidate C, simpatico employs a simple mechanism for accumulating moleculelevel support from atom-level embeddings.

To begin, embeddings $\vec{c_{n,j}}$ are computed for all atoms $c_{n,j}$ belonging to compounds in $C_{n}\in C$. The $\vec{c_{n,j}}$ embeddings are placed in a vector database D (Faiss [Douze et al., 2025]) for rapid retrieval of near neighbors in embedding space. Given a single target protein pocket P: for each $\vec{p_{i}}\in \vec{P}$, the k-nearest-neighbors $N(\vec{p_{i}})$ from $\vec{C}$ are identified from the database D. An embedding similarity $s(p_{i},c_{j})$ for each $\vec{p_{i}}∼\vec{c_{j}}$ is captured as described in the next paragraph. For each candidate ligand $C_{n}$, the score $S(P,C_{n})$ is computed as the sum of similarities of all identified neighbors $\vec{p_{i}}∼\vec{c_{n,j}}$:

$$S(P,C_{n})=\sum_{c_{n,j}\in N(p_{i})}s(p_{i}∼c_{n,j})$$

A measure of similarity $s(p_{i,c_{j})}$ for an atom pair is computed by calculating the Euclidean (L2) distance of the pair, and transforming this distance relative to (i) the greatest distance among all candidates pairs and (ii) a noise factor. First, a maximum pairwise (Euclidean) distance is identified among a large sample of ($p_{i}≁c_{j}$) pairs: $D_{max}=\max_{i,j}{\left\|(\vec{p_{i}}≁\vec{c_{j}})\right\|}_{2}$. All pairwise distances are converted to similarities by subtracting the pair distance from $D_{max}:s^{\prime }(p_{i},c_{j})=D_{max}-{\left\|(\vec{p_{i}},\vec{c_{j}})\right\|}_{2}$. A result of this calculation is that the most distant ligand-atom pair will have a similarity of 0, and the pair with smallest distance will have a score close to $D_{max}$.

These similarity scores are then adjusted in an attempt to remove the influence of random atom-pair matches on the accumulated score of a pocket-ligand pair. Simpatico computes the distribution of pairwise (Euclidean) distances between non-interacting ($p_{i}≁c_{j}$) atoms, and a noise threshold value t is identified such that 99% of all non-interacting pairs ($p_{i}≁c_{j}$) have $s^{\prime }(p_{i},c_{j})<t$. This value is then subtracted from all $s^{\prime }(p_{i},c_{j})$ values, with any resulting negative values clamped to zero: $s(p_{i},c_{j})=\max\{0,s^{\prime }(p_{i},c_{j})-0\}$.

## 3 RESULTS

### 3.1 Virtual screening experiments

The intended function of simpatico is to rapidly and accurately identify good candidate drugs for a target protein pocket. In general, this will be achieved by using simpatico to assign scores to compounds in a large database, and to treat high-scoring compounds as good drug candidates. Note that simpatico does not produce a predicted docked structure – it simply assigns scores to good candidates, with the intention that some other (slower) tool will be used to perform docking on a candidate pool that is substantially enriched by simpatico for active molecules.

We evaluated simpatico on three datasets that contain target proteins, known active ligands for those proteins, and a large number of ‘decoy’ compounds that (a) are either known or expected to have no binding activity with the target protein and (b) are intended to be difficult to distinguish from actives by virtue of sharing similar properties with the actives. We also tested simpatico in a massively-high-throughput screening test involving a target database of 600 million candidates.

### 3.2 Enrichment Factor

To evaluate the efficacy of simpatico as a binding activity filter, we measure the extent to which it can filter away large amounts of a target database without filtering out the active molecules in that database. In practice, if simpatico can reliably produce small, strongly enriched samples from much larger datasets, it can enable an accurate overall workflow by limiting compute-heavy work to only the small enriched dataset. The *enrichment factor* of a sample is a measure of how many more-than-expected active molecules remain after down-sampling a database. Specifically: for a target protein, a compound dataset S is composed of actives A and negatives N, so S=A∪N. Simpatico assigns scores to each compound, and the top d-percent scoring entries from S can be retained in a set called $S_{d}$. Let $A_{d}=\{a|a\in A\;\text{and}\;a\in S_{d}\}$. The enrichment factor of S at d% is:

$$EF_{d}(S)=\frac{|A_{d}|∕|A|}{|S_{d}|∕|S|}$$

### 3.3 High Throughput Virtual Screen

We expect that the greatest strength of simpatico lies in its speed, since rapid reduction of a database will enable the use of slower tools that (a) can produce docked predictions and (b) are likely in time to become more accurate. The use of embedding nearest neighbors, enabled by the use of the Faiss vector database library [Douze et al., 2025], allows simpatico to completely ignore atomic embeddings that have little chance of interacting with the protein-pocket target, thereby enabling efficient search over massive chemical databases. To assess simpatico’s utility in this area, we perform a virtual screen using the target actives from DUD-E, mixed with 600 million size-matched compounds from the Enamine REAL database of commercially available compounds [Enamine, 2022].

To construct our virtual screening database, we randomly selected 600 million compounds from the REAL database collection of ~ 2 billion compounds containing 29-38 heavy atoms. To avoid biased results in which the model simply selects compounds based on size, we filtered the actives for each DUD-E target to only include compounds of that size range, leaving 99 targets and 11,686 active molecules. Simpatico atomic embeddings for all actives and all sampled Enamine compounds were generated and sharded across 6000 separate Faiss library indexes, each containing embeddings for ~100,000 compounds (empirically, this was a number of ligands whose atom embeddings would fit in the 48GB RAM of the system’s NVIDIA L40S card).

Virtual screening was performed according to the scoring protocol described above. A single $D_{max}$ and noise threshold t was determined from the first shard of 100,000 embeddings, then used across all other shards. Each protein atom gathered the 2048 nearest ligand atoms in each shard, resulting in a total of 12.3M nearest neighbor ligand atoms for each protein atom. Protein-compound scores $S(P,C_{n})$ are computed for each compound $C_{n}$ by summing over supporting atoms, as described in Section 2.8.

We evaluated enrichment factors at down-sampling values of 1%, .01% and 0.0001%, corresponding to the top 6M, 60K, and 600 scoring molecules of the ~600M molecules screened respectively. Run time for search of the ~600M ligands for matches to a single protein was 2.5 hours; when all 99 proteins were searched as a batch, the highly parallel search completed in 9.2 hours.

For most targets, there are no actives among the top several hundred or thousand scoring compounds. However, there are several targets for which simpatico performed exceedingly well. For example, of the 47 actives corresponding to protein target PUR2, 32 were among the 617 top-scoring of all ~600M compounds. See supplementary material for a detailed report of the HTVS results.

### 3.4 Measuring efficacy with challenging decoy databases

Decoy databases like DEKOIS [Bauer et al., 2013], DUD-E [Eberhardt et al., 2021], and LIT-PCBA [Tran-Nguyen et al., 2020] provide collections of proteins with a combination of known active compounds and challenging decoy compounds. An important feature of the decoys is that they are not merely a set of randomly sampled molecules – they are either chemically similar to the actives or plausible binding candidates by some other measure.

#### 3.4.1 DEKOIS results

The DEKOIS data set contains 81 protein targets. For each target, DEKOIS provides 40 known actives paired with 1200 property-matched decoys, yielding a total of 97,200 decoy molecules across the full set.

**Figure 3:**
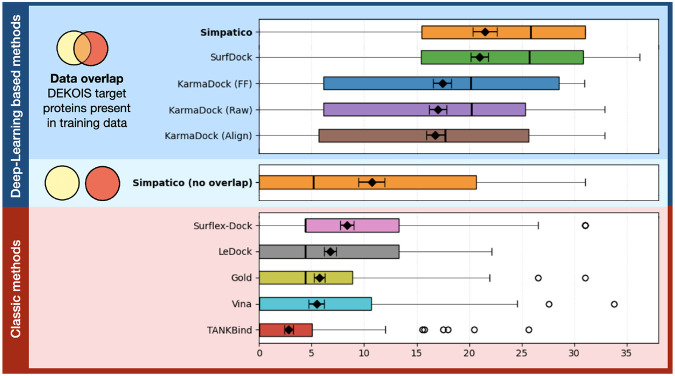
On the DEKOIS decoy benchmark, down-sampling to the 0.5% top-scoring candidates, enrichment factors for simpatico are state-of-art when compared to various docking and diffusion based complex-prediction models. Evaluation of the DEKOIS dataset was completed by simpatico in ~12 seconds.

We generated atomic embeddings for all DEKOIS protein pocket structures, and for all molecular compounds (active and decoy). Simpatico results are shown for models trained on two training subsets: one with DEKOIS protein targets present in the training data (but exact matching complexes removed), and another with all proteins in DEKOIS excluded from the training data (referred to as **Simpatico (no overlap)** in Figure 3). Scores for all actives and decoys were produced using the scoring function described above. Results for other modern open-source docking (pink) and Deep Learning-based (blue) methods were taken from Cao et al. [2025]. The enrichment factor at multiple down-sampling rates was then calculated for the complete set of scores; Figure 3 shows the results when considering the 0.5% top-scoring candidates, and is representative of relative results for other levels of down-sampling (see Supplemental Material). We highlight that these enrichment factors are expected to be very conservative estimates of the enrichment observed during generic screening, since the benchmark is designed to challenge the tool with inactive molecules that have been matched to the active molecules; high throughput virtual screens will contain many more easily-distinguished molecules. When trained on proteins included in the screen, Simpatico produced average enrichment factors of 20.4 and 21.3 at 1% and 0.5% down-sampling, respectively. Note that all Deep Learning approaches were trained on the full PDBBind (without filtering samples found in the test set). When DEKOIS protein targets were withheld from the training set (entailing a 20% reduction in training data), the model achieved average enrichment factors of 9.9 and 10.7 at 1% and 0.5% down-sampling. These results demonstrate (1) that simpatico learns features of drug interaction potential that generalize across protein pockets, and (2) when the training set includes ligand complexes for a given pocket, simpatico increases in ability to predict *other* ligands for that pocket. Simpatico required only ~12 seconds to perform the screening of the entire DEKOIS database.

#### 3.4.2 DUD-E and LIT-PCBA

We supplemented DEKOIS analysis with an exploration of enrichment on the DUD-E (Table 2) and LIT-PCBA (Supplemental Material) benchmarks. The mean observed enrichment factors (in the 1% top-scoring ligands for each protein) are much greater than classical docking approaches and are competitive with state of art tools based on diffusion or deep learning-based pose rescoring. Simpatico’s run times are at least 25,000x faster than competing methods run on a single core. [Note to reviewers: ^1^].

### 3.5 Other applications of atomic embeddings

Using a protein pocket embedding to query a database of small compounds is only one way to use Simpatico embeddings. In practice, there is no restriction on modality, as both small compound and protein pocket representations are projected into the same embedding space. We briefly explore two alternative ways to make use of atomic embeddings, with suggestive anecdotes for each case.

#### Ligand-Pocket Screen (toxicology)

One use case is to invert the search polarity, by creating a database of protein pockets, and searching that database with a query ligand. This could serve a useful purpose in toxicology or environmental impact studies, by suggesting candidates for possible undesirable binding interactions.

To investigate this use case, we identified three drug compounds known to bind with multiple non-homologous protein targets, the complexes of which have been resolved and made available through the Protein Database. These compounds included ritonavir, suramin, and ibuprofen.

We compiled a target database of all 102 protein pocket embeddings from the full DUD-E dataset, and appended the six known binding partners of the three drugs of interest. For each drug, we performed virtual drug-to-pocket screen per the previously described virtual screening protocol, querying the pocket collection with the chosen drug compounds. Instead of enrichment, we sorted the pocket scores and provide their rank as the metric. Table 5 shows that, for Ritonivir and Suramin, the two known binding partners were in the top 5 and top 2 (respectively) among all 107 candidate proteins. Disappointingly, Ibuprofen atoms failed to find any atom embedding matches for its target proteins, suggesting that these proteins are outside of the realm of simpatico training distribution.

#### Pocket Similarity Search

Another possible use case is the identification of proteins that have binding site pockets with similar binding potential. This might provide a mechanism for identifying proteins with similar function, even when sequence comparison provides no signal. Using the same protein database described in the previous section, we used each protein pocket as query, seeking other proteins with similar pocket. Table 4 shows results, for example indicating that 3p6h was the 7th best-scoring match (among 106) for the query pocket 2bxg. These results suggest the potential for atom embeddings that may arise with improved generality of embedding model (e.g. from more training data) and better score accumulation.

## 4 DISCUSSION

We have introduced a novel strategy for identifying promising candidates for protein-ligand binding activity. Classical docking approaches and newer diffusion-based methods require a great deal of computational power, restricting their application on billion-scale search. Simpatico re-casts the problem as a Representation Learning task based on a new “positive margin” loss function, and demonstrates remarkable speed gains over common methods. Simpatico’s GNN is fairly simple, and its simplistic summation score accumulation method is admittedly naive (for example, allowing physically incompatible atom pairings); even so, it is able to produce relatively high predictive accuracy.

We anticipate that large advances will result from improved models, better training data (for example using [Wang et al., 2025], or expanding to include molecular dynamics data from [Roy et al., 2025]), and schemes for accumulating support from atom pairs that improve on the simplistic approach currently employed in simpatico. We also note that we see promising improvements in both speed and accuracy in early tests in which we employ an HNSW database such as CAGRA [Ootomo et al., 2024] for Faiss-based near neighbor search.

To our knowledge, all of the learning-based models to which we compare simpatico decoy screening results were trained on a subset of PDBBind that also contained protein targets present in DUD-E, LIT-PCBA, and/or DEKOIS. Therefore, in order to make a fair comparison against these tools, we also trained an instance of our model on a subset of PDBBind that did not exclude proteins present in the screening task. This overlap in training/testing data is in fact consistent with what may be the strongest use case for simpatico: finding new ligands for pockets with some already-known ligands. For many protein targets, resolved protein-ligand complexes are already available; if not, there exist methods for generating denovo sets of plausible ligands, along with binding poses [Tang et al., 2024]. When such data is available, fine-tuning the model on these datasets may be preferable before embarking on a high-throughput virtual screen. Training on proteins present in DEKOIS emulates this fine-tuning step.

We also performed a separate training run such that the model would have no exposure to proteins present in the DEKOIS dataset. This has the added benefit of providing a “fairer” comparison with non-learning-based docking methods. While the model’s performance is significantly degraded relative to its fine-tuned counterpart, simpatico still demonstrates predictive accuracy greater than traditional docking methods, along with the aforementioned speed gains. Note that, though traditional docking methods were not trained on PDBBind/benchmark data in the same way as Deep Learning approaches, their parameters were presumably optimized to perform well on the benchmarks.

It should be noted that removing DEKOIS protein targets from the training set resulted in a significant reduction of training data, by ~ 20%. As such, the lesser performance of this model version is at least in part explained by having significantly less training data.

## Supplementary Material

1

## Figures and Tables

**Table 1:** HTVS results

	1% (R=6M)	0.01% (R=60K)	0.0001% (R=600)
EF (Enrichment Factor)	41.3	1020.5	25,985.9
Total # actives retained (among all 99*R top-scoring candidates)	5241	713	141

**Table 2:** DUD-E (102 proteins / 22,886 actives / 1,144,300 decoys)

	Vina	Gnina	Simpatico	PIGNet2	GenScore	RTMScore
EF_1_%	8.82	20.40	30.39	31.20	33.96	35.10
time	>5 hours^1^	>5 hours	26.5 secs	>5 hours	>5 hours	>5 hours

**Table 3:** Ligand-Pocket Screening Scores. Lower rank indicates higher score given small-molecule atom embed queries. No rank (−) indicates that the target pocket did not satisfy the scoring threshold, therefore receiving a score of 0.

Query	Target	Score
Ibuprofen	2bxg	-
Ibuprofen	3p6h	-
Ritonavir	4eyr	1
Ritonavir	5vce	5
Suramin	2h9t	2
Suramin	7ah8	1

**Table 4:** Pocket-Pocket Screening Scores

Query	Target	Score
2bxg	3p6h	7
3p6h	2bxg	7
5vce	4eyr	2
4eyr	7ah8	36
7ah8	2h9t	1
2h9t	7ah8	1

**Table 5:** Pocket-Pocket Screening Scores. Lower rank indicates higher score given pocket-pocket atom embed queries.

## Footnotes

1 EF values are taken from [Lam et al., 2024] and run times for non-simpatico tools are approximated by generously assuming only 1 second per comparison (which appears to be an underestimate for all tools) and perfect scaling across 64 cores. We are currently performing run time experiments with these tools (and reproducing EF results), but the results will not be ready in time for submission. We will add results in a camera-ready version of the paper.
