# Supplementary material for: Simpatico: accurate and ultra-fast virtual drug screening with atomic embeddings
Source: bioRxiv. 2025 Jun 8:2025.06.08.658499. Preprint. [Version 1] doi: 10.1101/2025.06.08.658499 (PMC12259003; doi:10.1101/2025.06.08.658499)
Supplement: 1 [file NIHPP2025.06.08.658499v1-supplement-1.pdf]

bioRxiv preprint doi: https://doi.org/10.1101/2025.06.08.658499; this version posted June 8, 2025. The copyright holder for this preprint (which was not certified by peer review) is the author/funder, who has granted bioRxiv a license to display the preprint in perpetuity. It is made available under aCC-BY 4.0 International license.

SUPPLEMENTARY MATERIAL

BENCHMARK DETAILS

Description of benchmark data sets (DEKOIS, DUD-E, and LIT-PCBA)

The DEKOIS [Bauer et al., 2013] data set contains 81 protein targets. For each target, DEKOIS provides 40 known actives paired with 1200 property-matched decoys, yielding a total of roughly 97,200 decoy molecules across the full set. DUD-E [Mysinger et al., 2012] includes 102 protein targets, with a total of 22,886 experimentally confirmed actives (an average of 224 actives per target). For each active ligand, 50 decoys are generated (similar in physico-chemical properties but dissimilar in topology), giving on the order of 1.14 million decoy compounds in total. LIT-PCBA [Tran-Nguyen et al., 2020] includes 15 distinct protein targets, each with experimentally confirmed actives and inactives. It contains a total of 7,761 true actives and 382,674 confirmed inactive compounds.

MODEL SPECIFICATIONS

Protein Encoder

The `ProteinEncoder` generates 64-dimensional embeddings for protein pocket surface atoms using a GAT-based residual architecture [Velickovic et al., 2017].

- **Input:** Each node has a 59-dimensional input feature vector, corresponding to a heavy (non-hydrogen) atom of the protein structure. Input vector indices correspond to the following features:
  - $[0 - 37]$: One-hot indicator of atom name, obtained from columns 13-16 in PDB file.
  - $[38 - 58]$: One-hot indicator of residue, obtained from columns 18-20 in PDB file.

  Atom features are projected to a 256-dimensional space (`hidden_dim` = 64, `heads` = 4).
- **Edge Construction:** Consider two atom types: *surface* and *other* (non-surface). For each type-pair, edges are assigned between the k nearest atoms according to the protein pocket structure.
  - surface – surface: $k = 15$
  - surface – other: $k = 15$
  - other – other: $k = 10$

  The set of other–other pairs is restricted: if a non-surface atom is among the 15 closest atoms to a surface atoms, an edge is established between itself and its 10 nearest neighbors. All unconnected atoms are eliminated from the pocket graph. Edges are assigned scalar edge weights via a 2-layer MLP applied to the concatenation of node features and inter-node distance.
- **Graph Layers:** The network uses 6 residual blocks (`blocks` = 6), each with 2 GATv2Conv layers (`block_depth` = 2). Each GATv2 layer operates on 256-dimensional features with 4 attention heads and uses scalar edge attributes. Outputs from each residual block are concatenated with the initial input.
- **Output:** The resulting 1792-dimensional concatenated representation is passed through a two-layer MLP with ReLU activation to produce 64-dimensional output embeddings (`out_dim` = 64) for surface atom nodes only.

`ProteinEncoder` returns surface atom embeddings along with their 3D coordinates and batch indices.

Small Molecule Encoder

The `MolEncoder` produces 64-dimensional atom embeddings from molecular graphs using stacked residual GATv2 layers with edge attributes.

- **Input:** Each small molecule heavy atom is represented by a 33-dimensional feature vector. Input vector indices correspond to the following features:

bioRxiv preprint doi: https://doi.org/10.1101/2025.06.08.658499; this version posted June 8, 2025. The copyright holder for this preprint (which was not certified by peer review) is the author/funder, who has granted bioRxiv a license to display the preprint in perpetuity. It is made available under aCC-BY 4.0 International license.

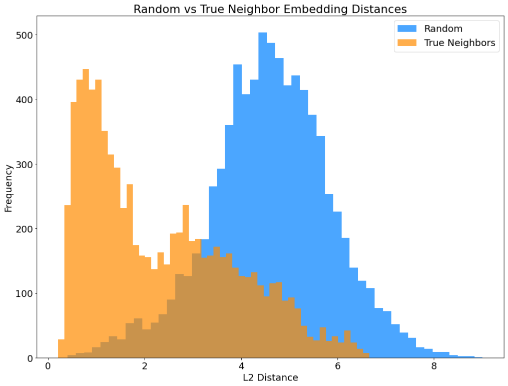

Figure S1: Random embeds vs neighbor embed distance

  - $[0 - 28]$: One-hot indicator of element name, according to RDKit naming conventions.
  - $[29 - 32]$: One-hot indicator of number of adjacent hydrogen atoms.

  Input vectors are projected to a 256-dimensional hidden space (`hidden_dim` = 64, `heads` = 4).

- **Edge Construction:** An edge is established between any two heavy atoms within 3 covalent bonds of one another.
- **Edge Attributes:** Each edge is represented by a 3-dimensional one-hot vector indicating topological distance in covalent-bond hops.
- **Graph Layers:** The encoder consists of 6 residual blocks (`blocks` = 6), each composed of 2 GATv2Conv layers (`block_depth` = 2), using 4 attention heads and incorporating the 3-dimensional edge attributes. Residual outputs from each block are concatenated with the initial input projection.
- **Output:** The concatenated tensor of shape 1792 ((`blocks + 1`) $\times$ `hidden_dim` $\times$ `heads`) is passed through a two-layer MLP with SiLU activation, yielding 64-dimensional final embeddings (`out_dim` = 64) for each atom.

`MolEncoder` returns an embedding vector for each atom in the input molecular graph.

TRAINING POCKET SPECIFICATION

During training, we use a semi-randomized method to generate the pocket graph. This is done to simulate conditions where we do not know precisely where a prospective 'ligand' will be located, thereby mitigating overfitting on a single pocket representation.

For each training sample, a *center* C is identified based on a randomly weighted combination of ligand atom coordinates. A 10-angstrom radius sphere of 1 angstrom resolution voxels is produced, centered on C. Therefore, the sphere is centered such that it generally contains the interaction sites between ligand and pocket, but is unlikely to capture the same set of atoms over subsequent batches.

Any voxel that is not 2 to 4 angstroms away from a protein atom is removed from the grid. The filtered voxel grid now represents a collection of points where we might plausibly expect to observe interactions with a ligand atom. For each of these points, the nearest protein atom is marked as a protein pocket surface atom. This is a heuristic approach to sampling the ligand-accessible protein atoms on the surface of the protein pocket.

ATOMIC EMBEDDING ANALYSIS

`Simpatico`'s GNN-based embedding strategy is intended to collocate embeddings for protein and compound atoms in high-dimensional space if they are physicochemically complementary, i.e. $p \sim c \implies \vec{p} \sim \vec{c}$. As an initial validation, we directly assessed `simpatico`'s ability to perform this training task by analyzing embedding distances between known binding partners and random atom embeds.

bioRxiv preprint doi: https://doi.org/10.1101/2025.06.08.658499; this version posted June 8, 2025. The copyright holder for this preprint (which was not certified by peer review) is the author/funder, who has granted bioRxiv a license to display the preprint in perpetuity. It is made available under aCC-BY 4.0 International license.

For each of the 102 protein targets in DUD-E, a docked protein-ligand complex is evaluated. Between all 102 of these complexes, there are 8107 protein-ligand atom pairs ($p_i \sim c_j$) within 4 angstroms of one another. We then measured the L2 embedding distance between each $\vec{p}$, $\vec{c}$ pair where $p \sim c$, as well as embedding distances between an equal number of random $\vec{p}$, $\vec{c}$ pairs from the DUDE protein-ligand complex data. The distributions of these distance measurements are illustrated in *Fig.* S1. The distribution of distances between a protein atom vector $\vec{p}$ and its interacting ligand partner vector $\vec{c_+}$ is significantly left-shifted relative to the distribution of distances between $\vec{p}$ and the vector for a random non-interacting compound atom $\vec{c_-}$ (mean values: 2.3 and 4.6, respectively).

ADDITIONAL BENCHMARK RESULTS

The LIT-PCBA benchmark

As in the analysis in Section 3.4.2, we analyzed the mean observed enrichment factor (in the 1% top-scoring ligands for each protein) for the LIT-PCBA benchmark. EF values are taken from [Lam et al., 2024] and run times for non-simpatico tools are approximated by generously assuming only 1 second per comparison (which appears to be an underestimate for all tools) and perfect scaling across 64 cores. [We are currently performing run time experiments with these tools (and reproducing EF results), but the results will not be ready in time for submission. We will add results in a camera-ready version of the paper].

Table S1: LIT-PCBA (15 proteins / 7,844 actives / 407,381 decoys)

	Vina	Gnina	Simpatico	GenScore	GRIM	IFP
$EF_{1\%}$	2.33	2.58	4.71	6.80	6.87	7.46
time	>1.8 hours	>1.8 hours	15.6 secs	>1.8 hours	>1.8 hours	>1.8 hours

Hit retention when filtering from very large datasets (high-throughput screening)

In Section 3.3, we showed that `simpatico` is capable of down-sampling a dataset containing 600 million decoys while producing a very high enrichment factor. When filtering a billion-compound library (where true binders are vanishingly rare), we suggest that the most critical statistic is the number of actives that survive the filter. This "true-hit retention" provides a direct assessment of screening utility and may be provide better intuition than reporting only enrichment factor (EF). To that effect, we present Table S2, which provides a sense of the number of families with various levels of retention at various levels of filtration. Specifically: for each filtration level leading to n remaining candidates, and each levels of hit retention r (ranging from 1 remaining true active to 100 actives), the table shows the number (out of 99 tested proteins) of target proteins for which at least r active ligands are found among the top n highest scoring ligands in the high throughput search.

Building a search index

For all benchmarks, the reported times correspond to the act of searching for atom-level neighbors in a pre-computed database. We provide the numbers in this way because we expect a typical use-case to involve offline index construction: on an infrequent basis, a large collection of candidate ligands is identified, embeddings are computed for all those ligands, and those embeddings are placed in

**Min Hits ≥**	**60M**	**600k**	**60k**	**6k**	**600**
1	84	67	41	25	16
2	80	61	35	20	11
5	75	48	26	16	7
10	68	41	20	12	5
25	56	26	10	4	2
50	31	13	4	1	0
100	17	2	1	0	0

Table S2: Number of targets with at least r actives among the top-scoring n molecules.

bioRxiv preprint doi: https://doi.org/10.1101/2025.06.08.658499; this version posted June 8, 2025. The copyright holder for this preprint (which was not certified by peer review) is the author/funder, who has granted bioRxiv a license to display the preprint in perpetuity. It is made available under aCC-BY 4.0 International license.

a vector database where they may be repeatedly used as a search index for many (very fast) future queries.

Even so, it is useful to understand the time required to produce the search indexes used in the various benchmarks. In general, whether using a single H100 GPU or all 96 cores of a Intel Xeon Platinum 8568Y+ chip with 1.2TB RAM, `simpatico` computes close to 100,000 ligand embeddings per minute, and over 1,000 protein pocket embeddings per minute. As a result, embedding plus Faiss database construction took roughly 12 minutes for the entire DUD-E preprocessing step. Even in an online search (in which all embeddings and search are performed for a single operation), the total run time (12.5 minutes) is much less than the >5 hours required for docking or diffusion approaches (Table 2).

Construction of the search index for the 600 million Enamine ligands required additional care, since the resulting Faiss database must fit in GPU RAM (48GB for the testin system's NVIDIA L40S card). As described in the main text, the collection of ligands was broken into 6000 shards, each containing $\sim$100,000 ligands. Total processing time to perform embedding and create the Faiss index was approximately 6 days.
